# Supplementary material for: Cytotoxic Properties of Polyurethane Foams for Biomedical Applications as a Function of Isocyanate Index
Source: Polymers (Basel). 2023 Jun 20;15(12):2754. doi: 10.3390/polym15122754 (PMC10301959; doi:10.3390/polym15122754)
Supplement: Supplementary file 1 [file polymers-15-02754-s001.zip › polymers-2381911-supplementary.pdf]

Supplementary Materials: Cytotoxic Properties of Polyurethane Foams for Biomedical Applications as a Function of Isocyanate Index

Dominik Grzęda[1,*], Grzegorz Węgrzyk[1], Adriana Nowak[2], Joanna Idaszek[1], Leonard Szczepkowski[3], Joanna Ryszkowska [1]

1 Faculty of Materials Science and Engineering, Warsaw University of Technology, Wołoska 141, 02-507 Warsaw, Poland; Dominik.grzeda.dokt@pw.edu.pl (D.G.); Grzegorz.wegrzyk.dokt@pw.edu.pl (G.W.); Joanna.ryszkowska@pw.edu.pl (J.R.); Joanna.idaszek@pw.edu.pl (J.I.)
2 Department of Environmental Biotechnology, Lodz University of Technology, Wolczanska 171/173, 90-530 Lodz, Poland; adriana.nowak@p.lodz.pl (A.N.)
3 Fampur Adam Przekurat leonardosz@interia.pl (L.S.)
* Correspondence: Dominik.grzeda.dokt@pw.edu.pl (D.G.);

Section S1. Foams for preliminary examinations

A series of PUF_2_0.6, PUF_2_0.7, PUF_2_0.8, and PUF_2_0.9 open-cell viscoelastic polyurethane foams were examined for preliminary cytotoxicity studies. Polyol masterbatches (Component A) were prepared by mixing substrates: polyols, catalysts, surfactant, and water according to a proprietary recipe from Fampur (Bydgoszcz, Poland). The foams in this series were made from component A with ROH=272 g/mol and isocyanate indices (INCO) = 0.6, 0.7, 0.8, and 0.9. The isocyanate component (B) was polymeric MDI (BorsodChem, Hungary), commercially traded as Ongronat 4040 TR, containing 32.6% of free isocyanate groups at a calculated equivalent value of RNCO = 128.8.

Section S2. Materials and Methodology of preliminary cytotoxicity studies

Cytotoxicity tests were performed according to ISO 10993-5. Extraction medium one and extraction medium two were used for the study.

Extraction medium one—culture medium (DMEM with L-alanine-L-glutamine and antibiotics (penicillin and streptomycin) without bovine serum (FBS) - extraction of hydrophilic compounds. Incubation for 24 h at 37 °C. FBS (10% v/v) was added at the start of incubation with cells.

Extraction medium two—culture medium (DMEM with L-alanine-L-glutamine and antibiotics (penicillin and streptomycin) with bovine serum (10% v/v) - extraction of hydrophilic and hydrophobic compounds. Incubation for 24 h at 37 °C. In experiment two, additional (5% v/v) FBS was added at the start of incubation with cells.

The foams were sterilized in an autoclave (121 °C, 30 min). In both cases, 0.1 g of sponge was used to obtain 1 ml of extract.

Mouse fibroblast cell line L929 (FSB).

Cells were seeded at a density of 1000 cells/well of a 96-well plate and cultured for 8h. Extracts were then added (1 type of extract to 10 wells) and incubated for 3 days (experiment one - E1 without FSB and E1 with ESB), and for 5 days (experiment two - E2 without FSB and E2 with ESB). The negative control is medium without and with FBS incubated under the same conditions as those with samples. Positive control: complete medium with 10% (v/v) DMSO added. After this time, cell viability was assessed using the MTS assay according to the manufacturer's recommendations (Promega).

Section 3. Results of preliminary cytotoxicity studies

Figure 1. shows the results of the cytotoxicity analysis of the foams.

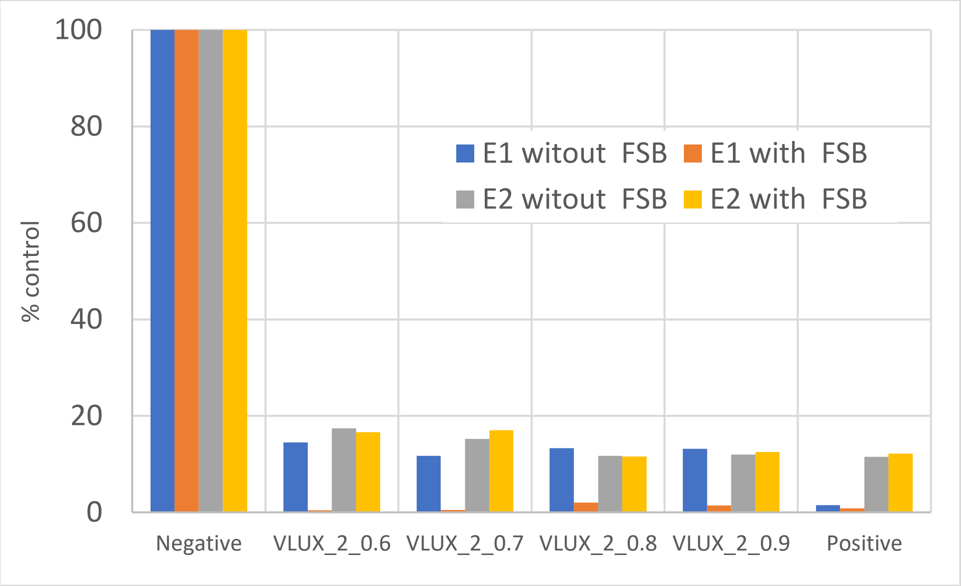

Figure 1 Results of foam cytotoxicity tests

For all tested foams, a decrease in the service life of more than 82% was observed (Fig 1). In tests conducted according to the proposed methods, a material that causes a decrease in lifespan by 70% compared to the negative control is considered toxic. The cytotoxic effect of compounds released from the foams was observed in both experiments. A stronger cytotoxic effect was observed in experiment No. 1 for the extraction medium with the addition of FBS, which could be due to the adsorption of proteins and growth factors present in FBS on the surface of the foams during incubation. This conjecture is supported by the fact that cytotoxicity decreased after adding an additive 5% (v/v) FBS to the extracts after incubation with the foams.
